# End-User Experience of the 2012 WHF Echocardiographic Criteria for Diagnosis of Rheumatic Heart Disease: A Global Survey

**DOI:** 10.5334/gh.1388

**Published:** 2025-01-27

**Authors:** Lene Thorup, Cleonice Mota, Krishna Kumar, Joselyn Rwebembera, James Marangou, Julius Chacha Mwita, Kate Ralston, Bo Reményi, Andrea Beaton, Liesl Zühlke, Ana Olga Mocumbi

**Affiliations:** 1 Department of Cardiothoracic Surgery, Rigshospitalet, Copenhagen University Hospital, Denmark; 2 Division of Paediatric and Fetal Cardiology, Department of Paediatrics, Universidade Federal de Minas Gerais, Belo Horizonte, Brazil; 3 Amrita Institute of Medical Sciences and Research Centre, Cochin, Kerala, India; 4 Uganda Heart Institute, Kampala, Uganda; 5 Menzies School of Health Research, Charles Darwin University, Darwin, Australia; 6 Royal Perth Hospital, Perth, Australia; 7 Fiona Stanley Hospital, Perth, Australia; 8 Department of Internal Medicine, University of Botswana and Princess Marina Hospital, Botswana; 9 World Heart Federation, Geneva, Switzerland; 10 Global and Tropical Health Division, Menzies School of Health Research, Charles Darwin University, Darwin, Australia; 11 Department of Paediatrics, Royal Darwin Hospital, Darwin, Australia and NT Cardiac, Darwin, Australia; 12 Department of Pediatrics, School of Medicine, University of Cincinnati, Cincinnati, Ohio, USA; 13 Division of Cardiology, The Heart Institute, Cincinnati Children’s Medical Center, Cincinnati, Ohio, USA; 14 Extramural Research & Internal Portfolio, South Africa Medical Research Council, South Africa; 15 Division of Paediatric Cardiology, Department of Paediatrics and Child Health, University of Cape Town, Cape Town, South Africa; 16 Universidade Eduardo Mondlane, Maputo, Mozambique; 17 Instituto Nacionall de Saúde, Marracuene, Mozambique

**Keywords:** Rheumatic Heart Disease, Echocardiography, Global Health

Early detection, adequate management, and initiation of secondary antibiotic prophylaxis to control rheumatic fever recurrences and improve rheumatic heart disease (RHD) outcome, are key in reducing the global burden of RHD (1). However, in endemic areas, RHD patients commonly present with advanced stages of disease (2,3), making management and treatment options challenging (4,5,6). Twelve years ago, the World Heart Federation (WHF) criteria for the echocardiographic screening and diagnosis of RHD (2012 WHF Criteria) (7) were developed, which has since been widely accepted as the standard for screening at risk populations. In 2022, considering the experience gained from 10 years of using these criteria, an evaluation was performed to i) assess the 2012 WHF criteria end-users’ perspectives on the challenges and limitations in criteria application, and ii) obtain feedback on the main features considered mandatory for future guidelines. The current end-user survey aimed at gathering feedback to support the revision of the updated criteria released in 2023 (8). The 23-question self-administered online survey contained both multiple-choice and open-ended answers, and was distributed through the network of Working Group members and through WHF channels and snowball sampling. The survey was in English and was developed by the working group based on personal experience with its application in diverse settings. Data was collected during four weeks in June 2022, was analyzed using R Studio (Core Team, 2021), and is presented as percentages. The study was exempted from bioethics committee approval.

**Figure 1:**
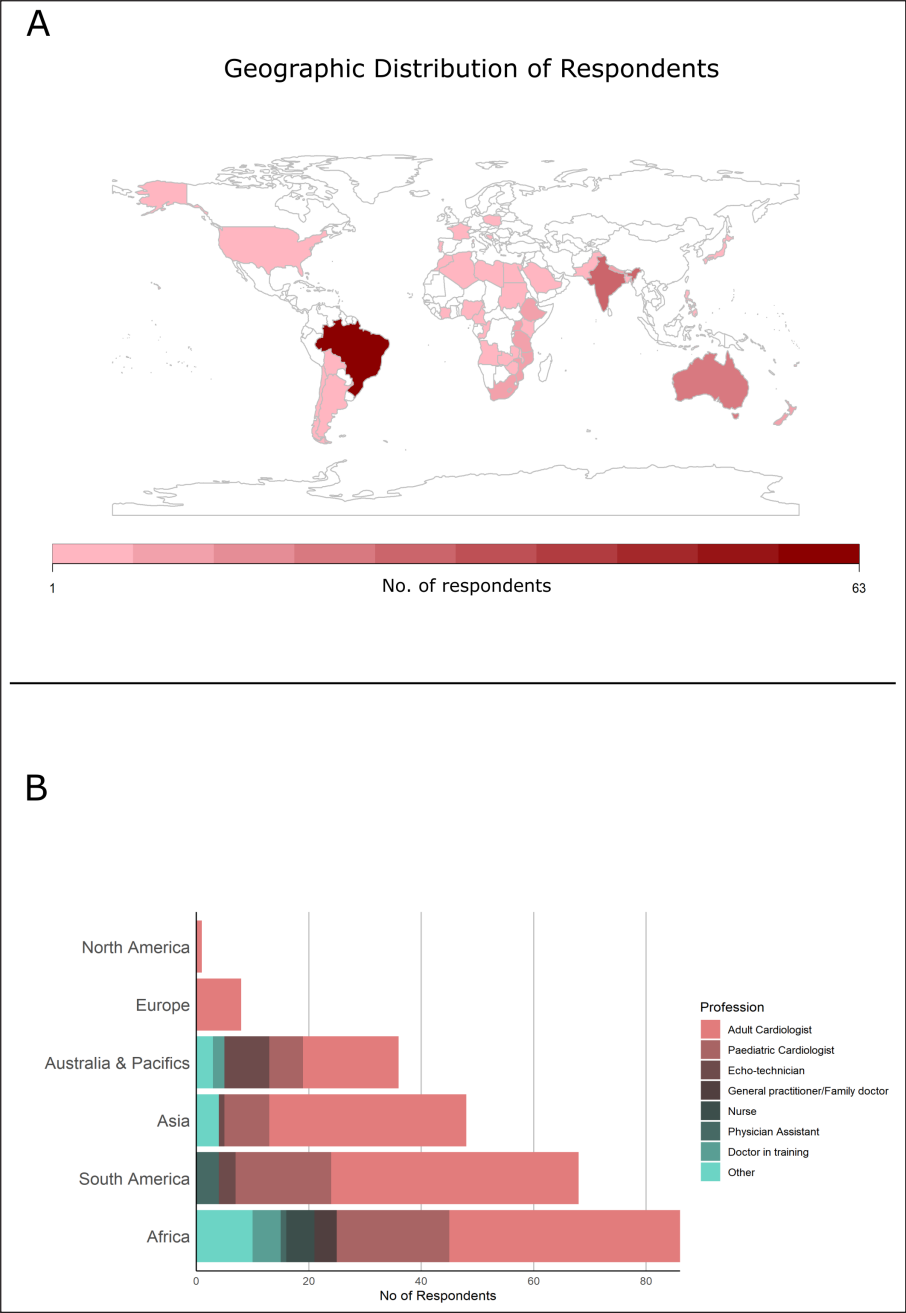
Distribution of respondents for the 2012 WHF criteria survey by country **(A)** and indicating the self-reported profession by region (n = 252) **(B)**.

The survey yielded responses from 302 participants, of which 33 were excluded due to blank answers. Thus, 269 participants were included in the analysis with varying number of responses for each question. Responses were received from 42 countries, with a higher contribution from RHD endemic areas (Figure 1). Of the 264 participants who stated their profession and location, 145 (54.9%) worked as adult cardiologists, 51 (19.3%) as pediatric cardiologists, with the remaining 25.8% being a mix of nurses, echo-technicians, junior doctors, general practitioners, cardiothoracic surgeons, and pediatricians with other sub-specialties. The percentage of trained cardiologists was 90% in Asia and South America, 71% in Africa, and 64% in Australia and the Pacific; all respondents from Europe (8) and North America (1) were cardiologists (Figure 1). In Australia and the Pacific, 8 out of 13 non-cardiologists were trained echo-technicians.

Among respondents, 81% (215/266) conducted screening for RHD either as routine practice or in large screening programs, and 86% (229/267) reported working in an endemic area for RHD. The survey revealed a high awareness (232/267, 87%) and application rate (215/227, 80%) of the 2012 WHF criteria. At this point, 35 respondents who were either unaware of or not applying the 2012 WHF criteria opted out of the survey. Of the remaining 234 respondents, 186 (79.5%) used the criteria for patient care, 64 (27%) for research purposes, and 71 (30.3%) for screening programs. Overall, users requested a simplified version of the criteria for screening (187, 79.9%), the inclusion of an algorithm to follow (131, 56.0%), an image library (76, 32.5%) and training programs for health workers (67, 28.6%). Throughout the survey, 54 (23.1%) end-users mentioned the morphological criteria and 17 (7.3%) the specific measurement of jet length/color Doppler features as valuable.

The limitations identified included the definition and interpretation of the “Borderline” category and the subjective nature of measuring some morphological criteria, as mentioned by 19 users. The use of colour Doppler/Continuous Wave Doppler was also found problematic, probably related to the fact that 70 (29.9%) of respondents experienced limited access to ultrasound equipment.

These survey results suggests that the 2012 WHF criteria were globally accepted as a tool for screening and diagnosing RHD. While intended to improve identification of RHD in asymptomatic people, the guidelines also strengthened reporting of RHD burden globally and supported research activities in endemic areas. Additionally, the guidelines’ application extended to patient care.

Most respondents were from RHD endemic regions with a high predominance of adult cardiologists. This is in accordance with the known lower availability of pediatric cardiologists (9) and the lacking involvement of other specialists in RHD care, despite this condition being a disease of childhood and adolescence in low- and middle-income countries. This discordance between preponderance of children and adolescents affected and adult cardiologists calls for the involvement of specialists and non-physicians in the management of high-risk populations such as children, adolescents, and women of reproductive age in RHD screening. In addition, multidisciplinary management teams should be established including pediatricians, physicians, family doctors, reproductive health specialists, general practitioners, and trained mid-level professionals – such as clinical officers and nurses trained for maternal and child health care. Indeed, task shifting to mid-level providers is expected to enable more widespread screening with comparable results to those obtained by specialists (10).

A key take-home message from the survey was the desire for an abbreviated set of criteria. This may be related to the sparse availability of trained cardiologists in most endemic settings, along with the challenges linked to using the criteria in settings with limited access to up-to-date echocardiographic equipment. In this context, identifying valvular lesions by a simplified set of criteria could improve early identification of RHD and ensure individuals are linked into the necessary care.

As the working group consists of a high number of cardiologist and physicians working in cardiology, the distribution of the survey through their networks led to a high percentage of cardiologists responding. This higher number of cardiology trained respondents probably skewed the results towards a more positive perception of the 2012 criteria, which may not reflect the actual criteria application experience by non-cardiology trained health professionals. This suspicion is supported by the requests of application and training features for the new criteria, along with the challenges of evaluating the subjective criteria.

In conclusion, the survey highlighted the need for a clearer definition of ‘Borderline’ disease, removal of subjective morphological assessments, and the addition of abbreviated criteria to be used in settings with limited access to state-of-the-art ultrasound equipment. The information obtained through this global survey was important to understand the end-users’ perspectives and was considered in the 2023 WHF criteria (8), especially the addition of abbreviated criteria for under-resourced areas endemic for RHD.
